# Effects of population age structure on parenteral antimicrobial use estimations

**DOI:** 10.1038/s41598-023-27769-z

**Published:** 2023-01-16

**Authors:** Ryuji Koizumi, Yoshiki Kusama, Yusuke Asai, Shinya Tsuzuki, Kensuke Aoyagi, Masahiro Ishikane, Yuichi Muraki, Norio Ohmagari

**Affiliations:** 1 AMR Clinical Reference Center, Disease Control and Prevention Center, National Center for Global Health and Medicine, 1-21-1 Toyama, Shinjuku-ku, Tokyo, 162-8655 Japan; 2 Department of Pediatric Infectious Diseases, Hyogo Prefectural Amagasaki General Medical Center, Amagasaki, Hyogo Japan; 3 Department of Clinical Pharmacoepidemiology, Kyoto Pharmaceutical University, Kyoto, Japan

**Keywords:** Diseases, Health care, Medical research

## Abstract

Antimicrobial use (AMU) is conventionally reported as unadjusted defined daily doses (DDDs) or population-adjusted DDDs per 1000 inhabitants per day (DID). DID is frequently used to monitor national AMU trends, this metric does not intrinsically take temporal changes in population age structure into account. We examined the effects of population age structure on DID estimates of parenteral AMU in Japan, and predicted future trends in DDDs based on population projections. Parenteral AMU data from 2013 to 2018 were acquired from a national claims database. We assessed temporal trends in parenteral AMU by age group (children aged < 15 years, working-age persons aged 15–64 years, and older persons aged ≥ 65 years) using both DID and DDDs. In addition, we modeled DDD predictions based on age-specific population projections from 2019 to 2030. DID values for older persons were 8.08–10.15 times and 5.43–5.63 times higher than in children and working-age persons, respectively. DID was stable, but DDDs increased in older persons. The prediction models showed that DDDs will continue to increase until 2030 if DID remains steady or decreases. DID estimates were skewed by the older population. More rigorous antimicrobial stewardship efforts targeting geriatric care are needed to counter the aging-associated increase in AMU.

## Introduction

Antimicrobial resistance (AMR) is a global health threat with potentially devastating medical and economic consequences, and requires the combined efforts of all countries to control its proliferation^1,2^. In response to this threat, the World Health Assembly endorsed a global action plan on AMR in which member states were requested to establish national action plans to prevent the further spread of resistant organisms^1^. As part of these plans, each country was tasked to monitor national antimicrobial use (AMU), which is a major contributor to AMR development^3^. Japan’s AMR national action plan was developed in 2016, and various countermeasures have been implemented by the government. These countermeasures include the publication of guidelines for appropriate oral AMU in upper respiratory infections and acute diarrhea in 2017, and the introduction of incentives for not prescribing antimicrobials to children aged < 3 years in 2018^4^.

A common metric of AMU is the number of defined daily doses (DDDs), which refers to the “assumed average maintenance dose per day for a drug used for its main indication in adults”^5–7^. The DDD is generally used to represent total unadjusted antimicrobial consumption, and is a more clinically relevant indicator than simple aggregations of administered quantities. However, a previous study has shown that parenteral AMU is not consistent across different age groups in Japan^8^. Furthermore, population adjustments can be incorporated by calculating DDDs per 1000 inhabitants per day (DID)^5^, which is the method employed by the World Health Organization and the European Centre for Disease Control and Prevention for presenting national AMU^5,6^.

In Japan, parenteral AMU calculated using DID showed no major changes from 2013 to 2018^9^. However, DID estimates do not take population age structure into account because this metric is calculated under the assumption that age distribution remains constant over time. As a consequence, DID comparisons across different time periods and groups can be problematic given real-world shifts in population composition. For example, population aging is a common issue among many developed societies^10,11^, and Japan currently has the most aged population in the world. Older persons tend to have higher rates of hospitalization, and therefore have more exposure to parenteral antimicrobials^12^. Despite the steady trends in DID in Japan^7^, the total consumption of parenteral antimicrobials in the Japanese population may actually have increased due to the rising proportion of older persons over time.

To examine the potential effects of population age structure on AMU estimates, we assessed the temporal trends in parenteral AMU in Japan from 2013 to 2018 according to three age groups (children, working age, and older persons) using both DID and DDDs. In addition, we predicted future trends in DDDs based on age-specific population projections from 2019 to 2030.

## Results

### Trends in parenteral AMU

Table 1 shows the population-adjusted parenteral AMU (DID) and total unadjusted parenteral AMU (DDDs) from 2013 to 2018 for the overall population and each age group (children aged < 15 years, working-age persons aged 15–64 years, and older persons aged ≥ 65 years). In the overall population, the DID and DDD values were higher in 2018 than in 2013. Among the age groups, the DID values for older persons were 8.1–10.2 times and 5.4–5.6 times higher than in children and working-age persons, respectively. Similarly, the DDD values for older persons were 16.3–26.3 times and 2.3–2.7 times higher than in children and working-age persons, respectively. Although the DID values for the three age categories had decreased from 2013 to 2018, the DID values for the total population had increased. This paradox could be explained by the fact that although the absolute amount of AMU (DDD) had increased due to population aging, the total population size had decreased during this period. Supplementary Table 1 presents the temporal changes in DID for frequently used antimicrobial agents.

**Table 1:** Parenteral antimicrobial use from 2013 to 2018 according to age group.

Age groups	2013	2014	2015	2016	2017	2018	Change from 2013 to 2018	Trends [95% CI]	*P*-value
Defined daily doses/1000 inhabitants/day									
< 15 years	0.25	0.24	0.25	0.24	0.22	0.2	− 0.05	− 0.03 [− 0.060, − 0.006]	0.03
15–64 years	0.37	0.36	0.37	0.37	0.36	0.36	− 0.01	− 0.01 [− 0.015, 0.003]	0.14
≥ 65 years	2.07	2	2.02	2.01	2.02	2.03	− 0.04	− 0.00 [− 0.010, 0.006]	0.57
Overall population	0.78	0.77	0.79	0.8	0.8	0.81	0.03	0.01 [0.003, 0.015]	< 0.01
Defined daily doses (million)									
< 15 years	1.48	1.4	1.43	1.39	1.24	1.14	− 0.34	− 0.04 [− 0.068, − 0.018]	< 0.01
15–64 years	10.65	10.3	10.41	10.38	9.96	9.78	− 0.87	− 0.01 [− 0.024, − 0.005]	0.02
≥ 65 years	24.06	24.05	25	25.46	25.97	26.32	2.26	0.02 [0.015, 0.027]	< 0.01
Overall population	36.2	35.75	36.85	37.23	37.17	37.24	1.04	0.01 [0.001, 0.015]	< 0.01

Trends are shown as the standardized coefficients from linear regression analysis of each metric from 2013 to 2018.

CI, confidence interval.

**Figure 1:**
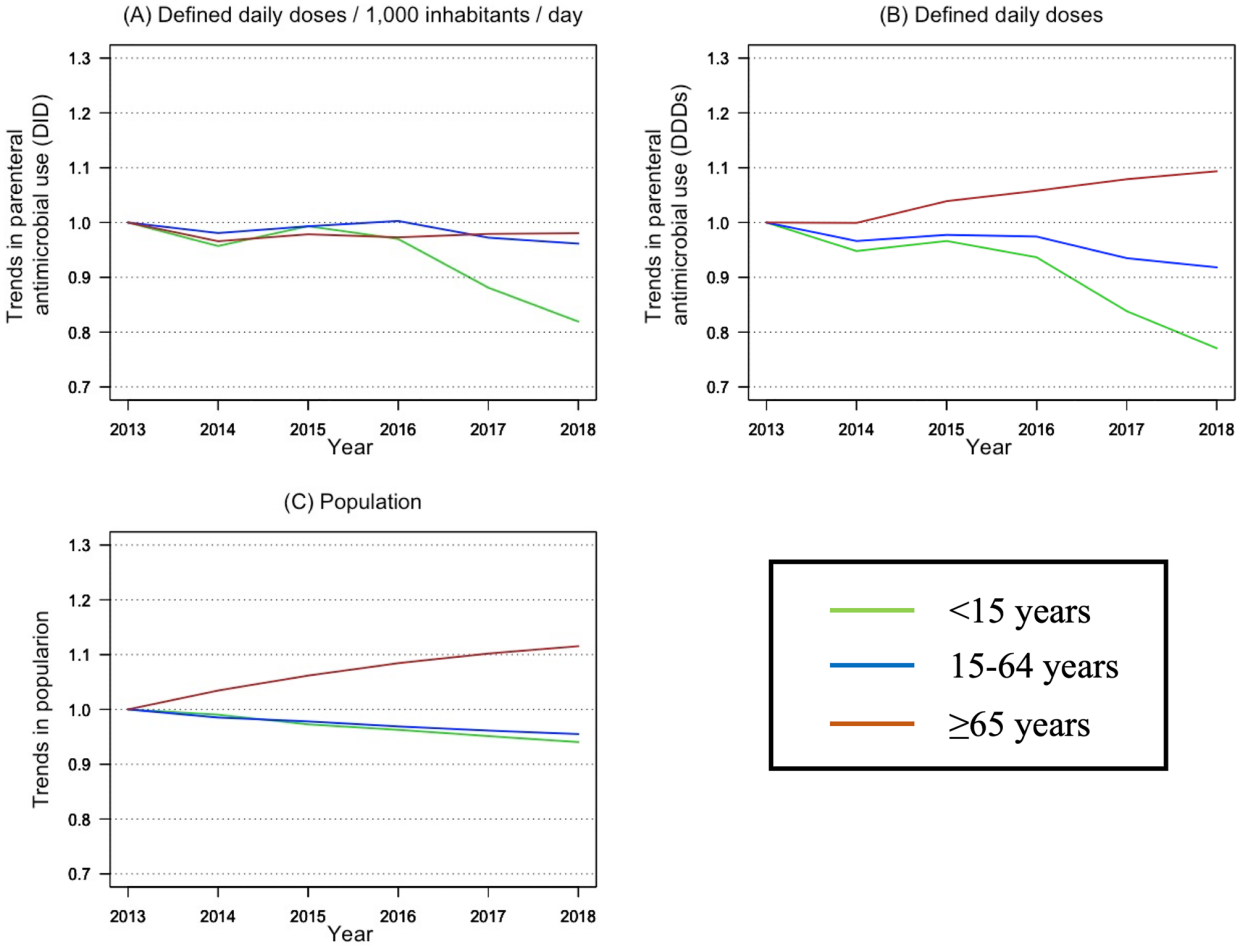
Trends in parenteral antimicrobial use and the national population from 2013 to 2018 according to age group. The graphs show the trends as ratios of each metric using the 2013 values as the reference points. (**A**) Trends in DID. (**B**) Trends in DDDs. (**C**) Trends in population numbers. DDD, defined daily dose; DID, defined daily doses/1000 inhabitants/day.

The age-specific trends in parenteral AMU and the national population from 2013 to 2018 are shown in Fig. 1 using the annual change rates relative to 2013 values. DID values decreased in children over the study period (*P* = 0.03) but were relatively stable in working-age persons (*P* = 0.14) and older persons (*P* = 0.57). In contrast, DDD values and the population decreased in children (*P* < 0.01) and working-age persons (*P* = 0.02) but showed upward trends in older persons (*P* < 0.01).

### Predictions of parenteral AMU

**Figure 2:**
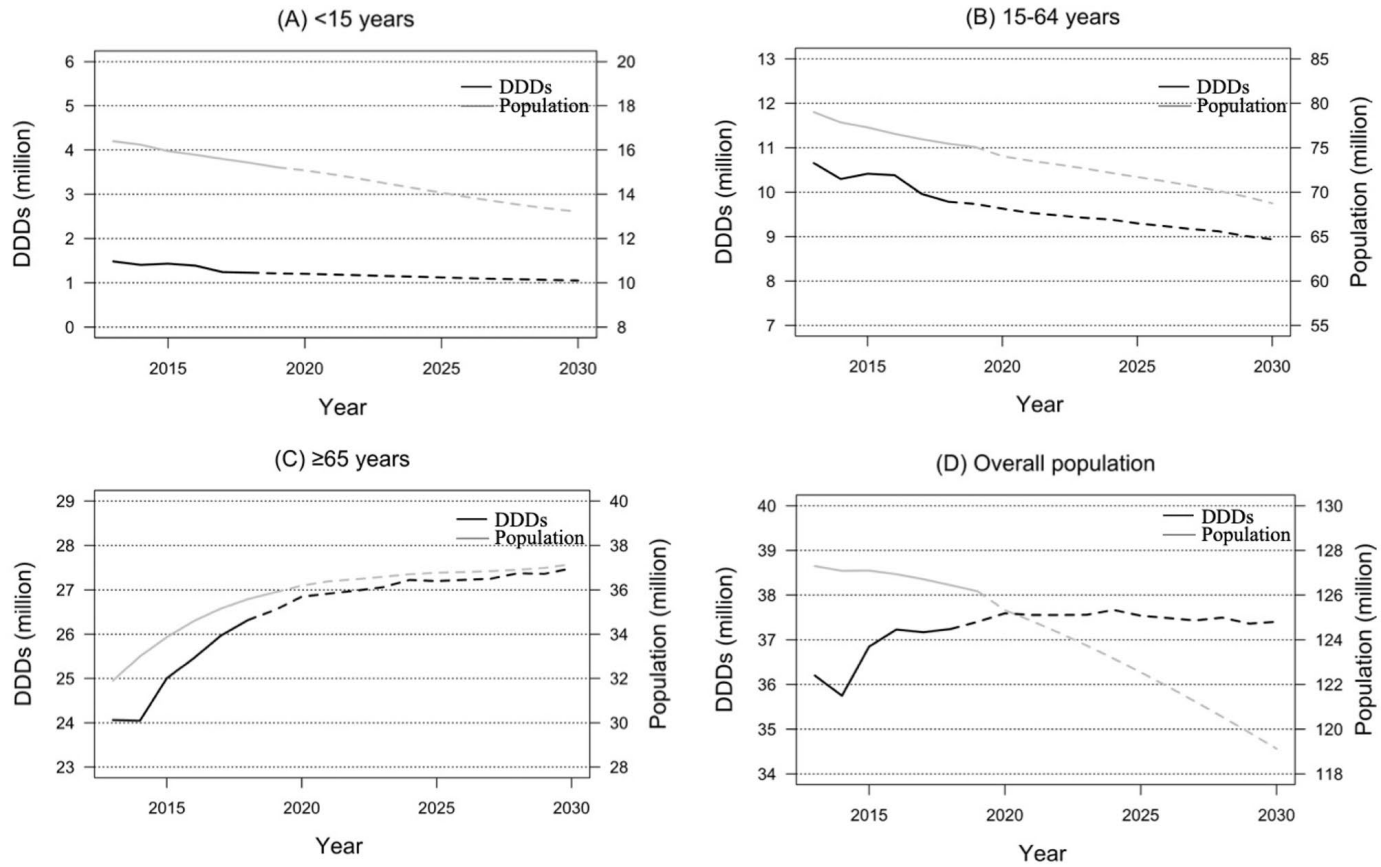
Trends and predictions in the DDDs of parenteral antimicrobials according to age group. (**A**) Children aged < 15 years. (**B**) Working-age persons aged 15–64 years. (**C**) Older persons aged ≥ 65 years. (**D**) Overall population. DDDs were predicted using the 2018 DID values. Solid lines represent chronological data of DDDs and population numbers from 2013 to 2018, and dashed lines represent the predicted values from 2019 to 2030 based on 2018 values. DDD, defined daily dose; DID, defined daily doses/1000 inhabitants/day.

Figure 2 presents the future predictions of DDDs in which DID was anchored at the 2018 value. For each age group, the predicted DDD values until 2030 generally changed in parallel with projected population changes. DDD values continued to decrease in children (*P* < 0.01) and working-age persons (*P* < 0.01), but increased in older persons (*P* < 0.01). However, when the age groups were aggregated, the predicted DDD values plateaued despite a decrease in total population (*P* = 0.115).

**Figure 3:**
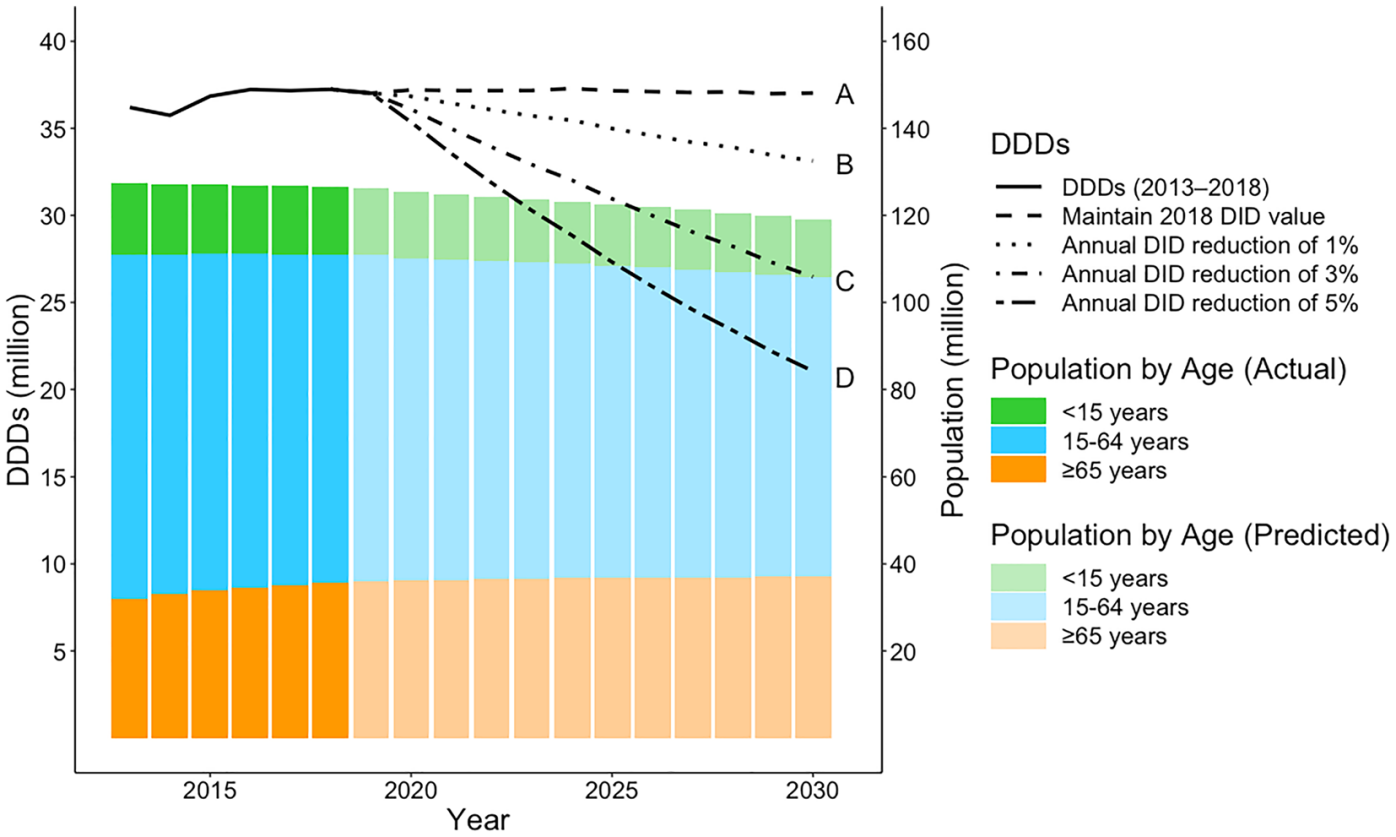
Scenario analysis of trends and predictions in the DDDs of parenteral antimicrobials according to annual DID reduction rate. Solid lines represent chronological data of DDDs. The dashed line A represents the predicted changes if DID maintains the 2018 value. The dotted line B represents the predicted changes if DID is decreased by 1% annually. The dash-dotted line C represents the predicted changes if DID is decreased by 3% annually. The dash-dotted line D represents the predicted changes if DID is decreased by 5% annually. The dark bars represent the actual population numbers, and the light bars represent the predicted population numbers. The population numbers are of the Japanese population^13,14^. DDD, defined daily dose; DID, defined daily doses/1000 inhabitants/day.

Figure 3 shows the results of the scenario analysis in which DDD predictions were made according to different annual DID reduction rates. DDD values were predicted to decrease in concordance with increasing levels of annual DID reduction (representing increasingly effective antimicrobial stewardship). The detailed results of this scenario analysis are provided in Supplementary Table 2. DDDs were projected to reach a 20% reduction by 2030 for an annual DID reduction of 2% (Table 2); however, this reduction could be achieved by 2024 for an annual DID reduction of 5%.

**Table 2:** Projected attainment of DDD reduction targets according to annual DID reduction rate.

	Target DDD value relative to 2018 value		
	95%	90%	80%
Maintain 2018 DID value	Unattainable	Unattainable	Unattainable
Annual DID reduction of 1%	2024	2029	Unattainable
Annual DID reduction of 2%	2021	2025	2030
Annual DID reduction of 3%	2020	2023	2027
Annual DID reduction of 4%	2020	2022	2025
Annual DID reduction of 5%	2019	2022	2024

DDD, defined daily dose; DID, defined daily doses/1000 inhabitants/day.

## Discussion

In this study, we used national-level health insurance claims data and population data to examine age-specific trends in parenteral AMU from 2013 to 2018 in Japan. Furthermore, we predicted the DDDs of parenteral AMU until 2030 based on population projections and different hypothetical levels of DID reductions. To the best of our knowledge, this is the first study that takes population shift into consideration for AMU changes. Our analysis showed that DID was stable in working-age persons and older persons, but decreased in children during the study period. In contrast, DDDs decreased in children and working-age persons together with population decline, but increased in older persons due to population aging. Overall DDD values were predicted to increase in older persons, but decrease in the younger age groups until 2030. In addition, DDD values were predicted to decrease with increasing DID reductions.

DID—as a population-adjusted metric of parenteral AMU—was found to be considerably higher in older persons than in the younger age groups. This indicates that the burden of AMU is disproportionately higher in older persons, and highlights the importance of antimicrobial stewardship initiatives that specifically target geriatric care. While inappropriate oral AMU for viral respiratory infections is a major issue for patients in all age categories^15^, the clinical context of parenteral AMU can vary according to age. For example, the morbidity of bacterial infections has decreased in Japanese children due to the widespread use of the *Haemophilus influenzae* type b vaccine and the polyvalent pneumococcal vaccine^16^. In contrast, aging populations have higher proportions of older persons who are more susceptible to bacterial infections^17^, as exemplified by the increasing pneumonia mortality in Japan over the past 30 years^18^. Therefore, the monitoring and reporting of parenteral AMU should be conducted according to age categories in addition to overall populations.

While DID is an important metric to analyze the impact of antimicrobial stewardship programs, its standardization to a population may overlook some important insights provided by analyzing total AMU. For example, increases in total antimicrobial consumption in a single hospital could potentially intensify antimicrobial pollution within an area, thereby exerting selective pressures that favor resistant bacterial lineages^19,17^. Moreover, excessive demand for antimicrobials may disrupt their supply and availability. Therefore, trends in DDDs for individual antibiotics should also be monitored in order to provide a more comprehensive understanding of AMU.

At the 2018 DID rate, our analysis predicted that DDDs in Japan will continue to increase until 2030 due to population aging. Accordingly, effective antimicrobial stewardship programs are needed to control the upward trends in AMU. Our models demonstrated that DID reduction can potentially decrease DDDs, with more aggressive DID reductions leading to faster results. These findings suggest a need to identify the subpopulations and health care settings with disproportionately high parenteral AMU, and implement targeted programs to reduce their use.

This study has several limitations. First, the NDB is a claims-based database, and as such lacks information on health care services not covered by health insurance (e.g., cosmetic surgeries and travelers’ healthcare) or those paid entirely by public funds. However, this is unlikely to have a substantial effect on our findings because those services form a relatively small proportion of total care given Japan’s universal health insurance coverage^20,21^. Second, our prediction models only considered changes in population age structure, and did not include other factors such as the incidences of infectious diseases. Incidence rates can be affected by public health conditions, distribution of vaccines, and infection control measures^22,23^. However, there were no major changes to these factors between 2013 and 2018. Third, government interventions implemented under Japan’s AMR national action plan may have impacted AMU in specific age groups. However, these interventions have focused on reducing oral AMU^4^, and would therefore have little impact on our study. Fourth, we had divided the study population into three age groups based on methods routinely used by Japan’s Ministry of Health, Labour and Welfare. Although the use of finer age categories may allow for a more thorough investigation, it would also increase the complexity of the analysis and interpretation of findings. Nevertheless, we consider a more detailed analysis to be the next step of this study.

## Conclusions

Population-adjusted parenteral AMU was substantially higher in older persons than in children and working-age persons from 2013 to 2018, and the temporal trends differed among these age groups. Monitoring DID according to age group may provide a better understanding of AMU trends. Total antimicrobial consumption is projected to increase in accordance with population aging. More rigorous antimicrobial stewardship efforts are needed to counter this aging-associated increase in AMU and prevent the spread of AMR.

## Methods

### Study data

This population-based retrospective study was conducted using the National Database of Health Insurance Claims and Specific Health Checkups of Japan (NDB). The NDB contains nationwide medical data from the Japanese health insurance system since April 2009^24,25^. The NDB contains data on all patients enrolled in any health insurance policy, but does not include data from public assistance recipients (approximately 1.7% of the Japanese population)^21^. National population age structure data from 2013 to 2019 were obtained from the Statistics Bureau, Ministry of Internal Affairs and Communications of Japan^13^. Population projections from 2020 to 2030 were acquired from the National Institute of Population and Social Security Research^14^, which provides three separate population projections calculated according to low, intermediate, and high birth rates and death rates. For this study, we used the population projection based on intermediate values (The differences in these three projections until 2030 were marginal)^14^.

From the NDB, we extracted parenteral AMU data from 2013 to 2018. We chose to analyze data from 2013 as Japan’s AMR national action plan was developed based on AMU data from 2013^4^. We did not include data from 2019 or later due to the occurrence of events that could have affected parenteral AMU in Japan, such as a cefazolin shortage and the COVID-19 pandemic^26,27^. Parenteral antimicrobials were identified using the World Health Organization’s Anatomical Therapeutic Chemical code J01^7^. To analyze the age-specific temporal trends in parenteral AMU, we divided the population into the following age groups: children (< 15 years), working-age persons (15–64 years), and older persons (≥ 65 years).

### Analysis

First, we described the temporal trends of parenteral AMU in Japan between 2013 and 2018 using DID (as an indicator of population-adjusted AMU) and DDDs (as an indicator of total unadjusted antimicrobial consumption). These metrics were calculated for the overall population as well as for each age group. We also calculated the annual change rates in DID, DDDs, and the national population as ratios relative to 2013 values. Linear regression analyses were conducted to calculate the 95% confidence intervals and *P* values of trends in AMU as time in years from 2013. Statistical significance was set at *P* < 0.05.

To clarify the effects of population shift on DDDs, we simulated DDD trends from 2019 until 2030 based on population projections. DDD predictions for parenteral AMU were modeled for the specific DID rate (in 2018) in each age group. In these models, DID was applied as a variable reflecting the effectiveness of antimicrobial stewardship interventions. DDDs were calculated using the following equations:

1 $$DDDs\left(t\right)={\sum }_{i}DD{Ds}_{i}(t) (1)$$

and

2 $$DD{Ds}_{i}(t)=DI{D}_{i}(t)\times 365 \;\;days\times Po{p}_{i}(t)/1000 (2)$$

where ${DDDs}_{i}(t)$, ${DID}_{i}\left(t\right)$ and ${Pop}_{i}(t)$ refer to the DDDs, DID, and population of age group *i* at year *t*, respectively.

DDDs until 2030 were calculated based on Japanese population projections using Eqs. (1) and (2) with DID set to the value in 2018, i.e., ${DID}_{i}\left(t\right)$ in Eq. (2) was fixed to ${DID}_{i}\left(2018\right)$.

Lastly, we conducted a scenario analysis to predict future DDD trends according to different levels of antimicrobial stewardship effectiveness, as indicated by the annual DID reduction rate. Such reductions were represented by a factor of $1-r\in [0, 1]$ with ${DID}_{i}(t)$ in Eq. (2) substituted by ${\left(1-r\right)}^{t-2018}{DID}_{i}(2018)$, meaning that ${DID}_{i}\left(t\right)$ decreases with reduction rate *r* annually from 2018. For this scenario analysis, we examined hypothetical annual DID reductions of 1%, 2%, 3%, 4%, and 5%. All analyses and numerical simulations were conducted using R software version 4.1.1 (R Core Team, 2017).

## Ethics declarations

This study was approved by the Institutional Review Board of the National Center for Global Health and Medicine (Approval number: NCGM-G-003098-00). Investigation was conducted according to the principles of the Declaration of Helsinki. Informed consents were waived because no patient information included in this study.

## Supplementary Information

Supplementary Tables.

## Data Availability

The data that support the findings of this study are available from the ministry of Health, Labour and Welfare of Japan but restrictions apply to the availability of these data, which were used under license for the current study, and so are not publicly available. Data are however available from the authors upon reasonable request and with permission of the ministry of Health, Labour and Welfare of Japan.
