# Supplementary material for: Effects of population age structure on parenteral antimicrobial use estimations
Source: Sci Rep. 2023 Jan 16;13:840. doi: 10.1038/s41598-023-27769-z (PMC9841941; doi:10.1038/s41598-023-27769-z)
Supplement: Supplementary file 1 — Supplementary Tables. [file 41598_2023_27769_MOESM1_ESM.docx]

Supplementary Table 1. DID of parenteral antimicrobials from 2013 to 2018 for 20 frequently used antimicrobial agents^a^

| ATC classification code | Antimicrobial agent | 2013 | 2014 | 2015 | 2016 | 2017 | 2018 | Change ratio  from 2013 to 2018 (%) |
| --- | --- | --- | --- | --- | --- | --- | --- | --- |
| J01DD04 | Ceftriaxone | 0.36 | 0.36 | 0.40 | 0.41 | 0.42 | 0.44 | 0.24 |
| J01DB04 | Cefazolin | 0.26 | 0.25 | 0.25 | 0.26 | 0.27 | 0.28 | 0.09 |
| J01CR01 | Ampicillin / sulbactam | 0.20 | 0.21 | 0.23 | 0.24 | 0.26 | 0.26 | 0.31 |
| J01CR05 | Piperacillin / tazobactam | 0.17 | 0.18 | 0.19 | 0.20 | 0.21 | 0.22 | 0.34 |
| J01DH02 | Meropenem | 0.11 | 0.12 | 0.12 | 0.13 | 0.13 | 0.13 | 0.15 |
| J01DC09 | Cefmetazole | 0.07 | 0.07 | 0.07 | 0.08 | 0.08 | 0.09 | 0.25 |
| J01MA12 | Levofloxacin | 0.06 | 0.06 | 0.06 | 0.06 | 0.06 | 0.06 | 0.11 |
| J01XA01 | Vancomycin | 0.05 | 0.05 | 0.05 | 0.05 | 0.05 | 0.05 | 0.49 |
| J01DC14 | Flomoxef | 0.08 | 0.07 | 0.06 | 0.05 | 0.05 | 0.04 | -0.48 |
| J01DD62 | Cefoperazone / sulbactam | 0.05 | 0.05 | 0.05 | 0.04 | 0.04 | 0.04 | -0.26 |
| J01DC07 | Cefotiam | 0.07 | 0.06 | 0.05 | 0.05 | 0.04 | 0.04 | -0.47 |
| J01AA08 | Minocycline | 0.06 | 0.05 | 0.05 | 0.04 | 0.04 | 0.04 | -0.32 |
| J01DE01 | Cefepime | 0.05 | 0.04 | 0.04 | 0.04 | 0.04 | 0.04 | -0.24 |
| J01FF01 | Clindamycin | 0.05 | 0.04 | 0.04 | 0.03 | 0.03 | 0.03 | -0.35 |
| J01GB11 | Isepamicin | 0.05 | 0.04 | 0.04 | 0.03 | 0.03 | 0.02 | -0.47 |
| J01DD02 | Ceftazidime | 0.04 | 0.03 | 0.03 | 0.02 | 0.02 | 0.02 | -0.38 |
| J01DH04 | Doripenem | 0.04 | 0.04 | 0.04 | 0.03 | 0.03 | 0.02 | -0.49 |
| J01CA01 | Ampicillin | 0.01 | 0.01 | 0.01 | 0.01 | 0.02 | 0.02 | 0.59 |
| J01DE03 | Cefozopran | 0.03 | 0.03 | 0.03 | 0.02 | 0.02 | 0.02 | -0.48 |
| J01DH51 | Imipenem / cilastatin | 0.03 | 0.03 | 0.02 | 0.02 | 0.02 | 0.02 | -0.52 |
| Other | Other antibiotics | 0.24 | 0.22 | 0.20 | 0.18 | 0.16 | 0.15 | -0.39 |

^a^20 most frequently used parenteral antimicrobial agents in older persons aged ≥65 years in 2018.

ATC, Anatomical Therapeutic Chemical; DID, defined daily doses / 1,000 inhabitants / day.

Supplementary Table 2. Scenario analysis of predictions in the DDDs of parenteral antimicrobials from 2019 to 2030 according to annual DID reduction rate

|  | | 2019 | | 2020 | | 2021 | | 2022 | | 2023 | | 2024 | | | 2025 | | 2026 | | 2027 | | 2028 | | 2029 | | 2030 | | Trend [95%Cl] | | | P-Value |
| --- | --- | --- | --- | --- | --- | --- | --- | --- | --- | --- | --- | --- | --- | --- | --- | --- | --- | --- | --- | --- | --- | --- | --- | --- | --- | --- | --- | --- | --- | --- |
| Maintain 2018 DID  value | | 37,403,246 (37,468,891  - 37,669,671) | | 37591527 (37,469,629  - 37,644,992) | | 37553673 (37,469,340  - 37,621,340) | | 37,552,816 (37,467,476  - 37,599,263) | | 37556716 (37,463,210  - 37,579,587) | | 37,667,600 (37,455,503  - 37,563,353) | | | 37,537,123 (37,443,532  - 37,551,382) | | 37,486,400 (37,427,298  - 37,543,675) | | 37,432,106 (37,407,622  - 37,539,409) | | 37,495,444 (37,385,545  - 37,537,545) | | 37,361,410 (37,361,893  - 37,537,256) | | 37,4032,48 (37,337,214  - 37,537,994) | | -11971 [-27430.5 , 3489.176] | | | 0.115 |
| Annual DID  reduction of 1% | | 37,029,213 (37,078,981  - 37,238,983) | | 36,843,455 (36,724,279  - 36,864,026) | | 36,438,291 (36,368,758  - 36,489,887) | | 36,073,085 (36,011,983  - 36,117,004) | | 35716063 (35,653,293  - 35,746,035) | | 35463297 (35,291,861  - 35,377,808) | | | 34987052 (34,927,032  - 35,012,978) | | 34590376 (34,558,805  - 34,651,546) | | 34194874 (34,187,835  - 34,292,857) | | 33910207 (33,814,952  - 33,936,081) | | 33451100 (33,440,814  - 33,580,561) | | 33132024 (33,065,857  - 33,225,859) | | -364829 [-377149.4 ,  -252509.5] | | | <0.01 |
| Annual DID reduction of 2% | | 36655181 (36,616,206  - 36,756,195) | | 36,102,902 (35,949,232  - 36,071,500) | | 35,345,217 (35,281,543  - 35,387,521) | | 34,637,522 (34,612,755  - 34,704,640) | | 33,948,297 (33,942,292  - 34,023,434) | | 33,367,556 (33,269,430  - 33,344,627) | | | 32,586,935 (32,593,596  - 32,668,792) | | 31,892,043 (31,914,789  - 31,995,930) | | 31,208,935 (31,233,582  - 31,325,468) | | 30,636,507 (30,550,701  - 30,656,680) | | 29,916,452 (29,866,722  - 29,988,990) | | 29,331,788 (29,182,027  - 29,322,016) | | -675834 [-6866135 ,  -665055.5] | | | <0.01 |
| Annual DID reduction of 3% | | 36,281,148 (36,009,988  - 36,314,552) | | 35,369,868 (35,079,664  - 35,345,674) | | 34,274,223 (34,147,784  - 34,378,354) | | 33,245,238 (33,213,514  - 33,413,423) | | 32,251,230 (32,275,601  - 32,452,135) | | 31,376,056 (31,332,468  - 31,496,067) | | | 30,329,351 (30,382,868  - 30,546,466) | | 29,379,717 (29,426,800  - 29,603,333) | | 28,457,050 (28,465,512  - 28,665,420) | | 27,650,045 (27,500,581  - 27,731,151) | | 26,724,669 (26,533,260  - 26,799,270) | | 25,935,011 (25564383  - 25868947) | | -949600 [-973051.6 ,  -926149.3] | | | <0.01 |
| Annual DID reduction of 4% | | 35,907,116 (35,308,606  - 35,880,657) | | 34,644,351 (34,155,302  - 34,654,937) | | 33,225,086 (32,999,073  - 33,432,143) | | 31,895,355 (31,838,356  - 32,213,837) | | 30,622,721 (30,670,796  - 31,002,373) | | 29,484,607 (29,493,432  - 29,800,713) | | | 28,207,176 (28,303,920  - 28,611,201) | | 27,042,298 (27,102,261  - 27,433,837) | | 25,923,006 (25,890,797  - 26,266,278) | | 24,928,195 (24,672,491  - 25,105,561) | | 23,845,521 (23,449,696  - 23,949,332) | | 22,902,370 (22,223,977  - 22,796,028) | | -1189512 [-1233559 ,  -1145464] | | | <0.01 |
| Annual DID reduction of 5% | | 35,533,084 (34,541,721  - 35,438,334) | | 33,926,353 (33,199,790  - 33,982,901) | | 32,197,580 (31,853,274  - 32,532,053) | | 30,587,003 (30,499,724  - 31,088,240) | | 29,060,671 (29,135,449  - 29,655,151) | | 27,689,147 (27,755,807  - 28,237,429) | | | 26,213,573 (26,357,125  - 26,838,747) | | 24,869,243 (24,939,403  - 25,459,105) | | 23,591,563 (23,506,314  - 24,094,830) | | 22,449,907 (22,062,500  - 22,741,280) | | 21,251,174 (20,611,653  - 21,394,764) | | 20,198,025 (19,156,220  - 20,052,833) | | -1398682 [-1467720 ,  -1329644] | | | <0.01 |
|  | 2019 | | 2020 | | 2021 | | 2022 | | 2023 | | 2024 | | 2025 | 2026 | | 2027 | | 2028 | | 2029 | | 2030 | | Trend [95%Cl] | | P-Value | |  |  |  |
|  |  |  |  |  |  |  |  |  |  |  |  |  |  |  |  |  |  |  |  |  |  |  |  |  |  |  |  |  |  |  |
| Maintain 2018 DID value | 37,403,246 | | 37591527 | | 37553673 | | 37,552,816 | | 37556716 | | 37,667,600 | | 37,537,123 | 37,486,400 | | 37,432,106 | | 37,495,444 | | 37,361,410 | | 37,4032,48 | | -11,971 | | 0.115 | |  |  |  |
| -Lower | 37,468,891 | | 37,469,629 | | 37,469,340 | | 37,467,476 | | 37,463,210 | | 37,455,503 | | 37,443,532 | 37,427,298 | | 37,407,622 | | 37,385,545 | | 37,361,893 | | 37,337,214 | | -27,430.50 | |  |  |  |  |  |
| -Upper | 37,669,671 | | 37,644,992 | | 37,621,340 | | 37,599,263 | | 37,579,587 | | 37,563,353 | | 37,551,382 | 37,543,675 | | 37,539,409 | | 37,537,545 | | 37,537,256 | | 37,537,994 | | 34,892 | |  |  |  |  |  |
| Annual DID reduction of 1% | 37,029,213 | | 36,843,455 | | 36,438,291 | | 36,073,085 | | 35716063 | | 35463297 | | 34987052 | 34590376 | | 34194874 | | 33910207 | | 33451100 | | 33132024 | | -364,829 | | <0.01 | |  |  |  |
| -Lower | 37,078,981 | | 36,724,279 | | 36,368,758 | | 36,011,983 | | 35,653,293 | | 35,291,861 | | 34,927,032 | 34,558,805 | | 34,187,835 | | 33,814,952 | | 33,440,814 | | 33,065,857 | | 377,149.40 | |  |  |  |  |  |
| -Upper | 37,238,983 | | 36,864,026 | | 36,489,887 | | 36,117,004 | | 35,746,035 | | 35,377,808 | | 35,012,978 | 34,651,546 | | 34,292,857 | | 33,936,081 | | 33,580,561 | | 33,225,859 | | 252,509.50 | |  |  |  |  |  |
| Annual DID reduction of 2% | 36655181 | | 36,102,902 | | 35,345,217 | | 34,637,522 | | 33,948,297 | | 33,367,556 | | 32,586,935 | 31,892,043 | | 31,208,935 | | 30,636,507 | | 29,916,452 | | 29,331,788 | | -675,834 | | <0.01 | |  |  |  |
| -Lower | 36,616,206 | | 35,949,232 | | 35,281,543 | | 34,612,755 | | 33,942,292 | | 33,269,430 | | 32,593,596 | 31,914,789 | | 31,233,582 | | 30,550,701 | | 29,866,722 | | 29,182,027 | | -686,613.50 | |  |  |  |  |  |
| -Upper | 36,756,195 | | 36,071,500 | | 35,387,521 | | 34,704,640 | | 34,023,434 | | 33,344,627 | | 32,668,792 | 31,995,930 | | 31,325,468 | | 30,656,680 | | 29,988,990 | | 29,322,016 | | -665,055.50 | |  |  |  |  |  |
| Annual DID reduction of 3% | 36,281,148 | | 35,369,868 | | 34,274,223 | | 33,245,238 | | 32,251,230 | | 31,376,056 | | 30,329,351 | 29,379,717 | | 28,457,050 | | 27,650,045 | | 26,724,669 | | 25,935,011 | | -949,600 | | <0.01 | |  |  |  |
| -Lower | 36,009,988 | | 35,079,664 | | 34,147,784 | | 33,213,514 | | 32,275,601 | | 31,332,468 | | 30,382,868 | 29,426,800 | | 28,465,512 | | 27,500,581 | | 26,533,260 | | 25564383 | | -973,051.60 | |  |  |  |  |  |
| -Upper | 36,314,552 | | 35,345,674 | | 34,378,354 | | 33,413,423 | | 32,452,135 | | 31,496,067 | | 30,546,466 | 29,603,333 | | 28,665,420 | | 27,731,151 | | 26,799,270 | | 25868947 | | -926,149.30 | |  |  |  |  |  |
| Annual DIDreduction of 4% | 35,907,116 | | 34,644,351 | | 33,225,086 | | 31,895,355 | | 30,622,721 | | 29,484,607 | | 28,207,176 | 27,042,298 | | 25,923,006 | | 24,928,195 | | 23,845,521 | | 22,902,370 | | -1,189,512 | | <0.01 | |  |  |  |
| -Lower | 35,308,606 | | 34,155,302 | | 32,999,073 | | 31,838,356 | | 30,670,796 | | 29,493,432 | | 28,303,920 | 27,102,261 | | 25,890,797 | | 24,672,491 | | 23,449,696 | | 22,223,977 | | -1,233,559 | |  |  |  |  |  |
| -Upper | 35,880,657 | | 34,654,937 | | 33,432,143 | | 32,213,837 | | 31,002,373 | | 29,800,713 | | 28,611,201 | 27,433,837 | | 26,266,278 | | 25,105,561 | | 23,949,332 | | 22,796,028 | | -1,145,464 | |  |  |  |  |  |
| Annual DIDreduction of 5% | 35,533,084 | | 33,926,353 | | 32,197,580 | | 30,587,003 | | 29,060,671 | | 27,689,147 | | 26,213,573 | 24,869,243 | | 23,591,563 | | 22,449,907 | | 21,251,174 | | 20,198,025 | | -1,398,682 | | <0.01 | |  |  |  |
| -Lower | 34,541,721 | | 33,199,790 | | 31,853,274 | | 30,499,724 | | 29,135,449 | | 27,755,807 | | 26,357,125 | 24,939,403 | | 23,506,314 | | 22,062,500 | | 20,611,653 | | 19,156,220 | | -1,467,720 | |  |  |  |  |  |
| -Upper | 35,438,334 | | 33,982,901 | | 32,532,053 | | 31,088,240 | | 29,655,151 | | 28,237,429 | | 26,838,747 | 25,459,105 | | 24,094,830 | | 22,741,280 | | 21,394,764 | | 20,052,833 | | -1,329,644 | |  |  |  |  |  |

DDD, defined daily dose; DID, defined daily doses / 1,000 inhabitants / day.

Parentheses indicate 95 ％confidence intervals
